# Presence and diversity of anammox bacteria in cold hydrocarbon-rich seeps and hydrothermal vent sediments of the Guaymas Basin

**DOI:** 10.3389/fmicb.2013.00219

**Published:** 2013-08-02

**Authors:** Lina Russ, Boran Kartal, Huub J. M. op den Camp, Martina Sollai, Julie Le Bruchec, Jean-Claude Caprais, Anne Godfroy, Jaap S. Sinninghe Damsté, Mike S. M. Jetten

**Affiliations:** ^1^Department of Ecological Microbiology, Institute for Wetland and Water Research, Radboud UniversityNijmegen, Netherlands; ^2^Department of Marine Organic Biogeochemistry, NIOZ Royal Netherlands Institute for Sea ResearchDen Burg, Texel, Netherlands; ^3^Unité Etude des Ecosystèmes Profonds, Laboratoire Environnement ProfondsIfremer, Plouzané, France; ^4^Unité Etude des Ecosystèmes Profonds, Laboratoire de Microbiologie des Environnements Extrêmes, Ifremer, UMR6197, CNRS, University of BrestPlouzané, France

**Keywords:** anammox, *hzsA*, *aprA*, hydrothermal vents, cold seep, sulfide

## Abstract

Hydrothermally active sediments are highly productive, chemosynthetic areas which are characterized by the rapid turnover of particulate organic matter under extreme conditions in which ammonia is liberated. These systems might be suitable habitats for anaerobic ammonium oxidizing (anammox) bacteria but this has not been investigated in detail. Here we report the diversity and abundance of anammox bacteria in sediments that seep cold hydrocarbon-rich fluids and hydrothermal vent areas of the Guaymas Basin in the Cortés Sea using the unique functional anammox marker gene, hydrazine synthase (*hzsA*). All clones retrieved were closely associated to the “*Candidatus* Scalindua” genus. Phylogenetic analysis revealed two distinct clusters of *hzsA* sequences (*Ca*. Scalindua *hzsA* cluster I and II). Comparison of individual sequences from both clusters showed that several of these sequences had a similarity as low as 76% on nucleotide level. Based on the analysis of this phylomarker, a very high interspecies diversity within the marine anammox group is apparent. Absolute numbers of anammox bacteria in the sediments samples were determined by amplification of a 257 bp fragment of the *hszA* gene in a qPCR assay. The results indicate that numbers of anammox bacteria are generally higher in cold hydrocarbon-rich sediments compared to the vent areas and the reference zone. Ladderanes, lipids unique to anammox bacteria were also detected in several of the sediment samples corroborating the *hzsA* analysis. Due to the high concentrations of reduced sulfur compounds and its potential impact on the cycling of nitrogen we aimed to get an indication about the key players in the oxidation of sulfide in the Guaymas Basin sediments using the alpha subunit of the adenosine-5′-phosphosulfate (APS) reductase (*aprA*). Amplification of the *aprA* gene revealed a high number of gammaproteobacterial *aprA* genes covering the two sulfur-oxidizing bacteria *aprA* lineages as well as sulfate-reducers.

## Introduction

The Guaymas Basin is a near-shore submarine depression in the central Gulf of California that is characterized by hydrothermally active sediments and hydrocarbon-rich seepages that escape from the sediments at a range of different temperatures (Bazylinski et al., 1988). Geothermally-heated seawater water rises along the ridge segment that is associated with sea floor spreading and escapes via hotspots at high temperatures up to 300°C (Von Damm et al., 1985). Locally, the progressive degradation of organic matter in deeper sediment layers causes the buildup of hydrogen sulfide, methane and other hydrocarbons that seep to the sediment surface at moderate temperatures (cold hydrocarbon-rich seeps) (Vigneron et al., 2013). Due to a high sedimentation rate of the detritus from the productive surface waters as well as terrestrial input, the crustal fissures are covered with a layer of 100–500 m-thick organic-rich sediments (Calvert, 1966). The hot fluids diffusing upwards lead to accelerated diagenesis by metal sulfide precipitation and thermochemical decomposition of organic material within the sediment, leading to a distinct seeping fluid with relatively higher concentrations of ammonium and low molecular weight hydrocarbons and lower concentrations of free sulfides than other lava vent sites (Kawka and Simoneit, 1987; Bazylinski et al., 1988; Von Damm, 1990). This creates an unusual ecosystem at 2000 m depth that is fueled by conversion of reduced inorganic compounds such as hydrogen sulfide or methane by chemoautotrophs. Although the Guaymas Basin sediment is generally well-supplied with ammonium (Von Damm, 1990) and there is sufficient evidence of nitrate reduction as a potential source for nitrite (Bowles and Joye, 2010; Bowles et al., 2012), anaerobic ammonium oxidation (anammox) has not yet been investigated in Guaymas Basin sediments. It has been assumed that the abundance of reduced carbon and sulfide may favor denitrification and dissimilatory nitrate reduction to ammonium (DNRA) and inhibit anammox (Burgin and Hamilton, 2007; Jensen et al., 2008).

Anammox has been shown to be a key process in the cycling of nitrogen in oxygen-limited systems such as oxygen minimum zones and marine sediments all over the world and molecular surveys could confirm presence of anammox bacteria in very diverse environments such as deep sea sediments, Atlantic hydrothermal vent systems, hot springs, arctic sediments, and petroleum reservoirs (Kuypers et al., 2003, 2005; Trimmer et al., 2003; Byrne et al., 2008; Jaeschke et al., 2009, 2010; Lam et al., 2009; Li et al., 2010; Hong et al., 2011; Harhangi et al., 2012; Borin et al., 2013). So far, anammox bacteria of the genus “*Candidatus* Scalindua spp.” are the major representatives of the order *Brocadiales* in marine ecosystems (van de Vossenberg et al., 2008, 2013; Woebken et al., 2008). Like other anammox bacteria, they derive their energy for growth from the conversion of NH^+^_4_ and NO^−^_2_ into dinitrogen gas, thereby constituting an important sink for fixed nitrogen under anoxic conditions. In this study we used a combination of specific biomarkers to target anammox bacteria and determine their numbers and diversity within the Guaymas basin. Anammox bacteria are so far the only known bacteria capable of hydrazine production, therefore we used the alpha subunit of the hydrazine synthase (HzsA) complex as a molecular marker to detect and quantify anammox (Harhangi et al., 2012). In addition we used ladderane lipids, which are unique to the membranes of anammox bacteria, as biomarkers. (Sinninghe Damsté et al., 2002, 2005).

The importance of the sulfur cycle in the Guaymas Basin sediments has been known already since the discovery of extensive mats of sulfur-oxidizing *Beggiatoa spp*. that were observed at the sediment interface (Jannasch et al., 1989). These organisms thrive on sulfide and nitrate and contribute significantly to the systems primary production (Nelson et al., 1989; McHatton et al., 1996). As the abundance of reduced sulfur compounds might have a substantial impact on the cycling of nitrogen in the Guaymas Basin sediments, we also investigated the diversity of the gene encoding a key enzyme of the dissimilatory sulfate-reduction pathway: dissimilatory adenosine-5′-phosphosulfate (APS) reductase. Homologues of this gene have been found in photo- and chemotrophic sulfur oxidizers, in which it is thought to work in reverse direction, converting sulfite to APS (Frigaard and Dahl, 2009). Two other groups of sulfur oxidizers have been found to be important in marine sediments using different pathways to oxidize reduced sulfur compounds: *Epsilonproteobacteria* using the Sox pathway and *Gammaproteobacteria* related to thiotrophic endosymbionts using the adenosine-5′-phosphosulfate pathway of sulfur oxidation (Hügler et al., 2010). The coupling of the conversion of reduced sulfur compounds to nitrate reduction could have very interesting implications with respect to the formation of complex interactions that would be fueled from the exchange of intermediates.

## Materials and methods

### Sample collection

Samples were recovered from cold hydrocarbon-rich seeps and hydrothermal vent sediments during the cruise “BIG” (RV L'Atalante, June 2010) on dives 1758-14, 1755-11, 1764-20, and 1766-22 as described by Vigneron et al. (2013). Sets of one location were sampled at about 12 cm distance. A description of the samples can be found in Table 1.

**Table 1:** **Description of the different samples used in this study**.

**No.**	**Dive**	**Core**	**(cm)**	**Location**	**Zone**	**Date**	**Depth (m)**	**Latitude**	**Longitude**	**References**
1	1758-14	CT2	0–3	Cold Seep, Vasconcelos active site, white mat	Sonora Margin	6/19/2010	1574	N 27 35.5750	W 111 28.9840	Vigneron et al., 2013
2	1758-14	CT2	3–6									
3	1758-14	CT2	6–9							
4	1758-14	CT2	9–12							
5	1758-14	CT2	12–15							
6	1758-14	CT2	15–18							
7	1758-14	CT2	18–21							
8	1758-14	CT1		Cold Seep, Vasconcelos active site, white mat	Sonora Margin	6/19/2010	1574	N 27 35.5754	W 111 28.9860	
9	1758-14	CT11	0–4	Cold Seep, Vasconcelos active site, edge of white mat	Sonora Margin	6/19/2010	1574	N 27 35.5872	W 111 28.9859	
10	1758-14	CT11	4–6							
11	1755-11	CT1		Cold seep, Vasconcelos active site	Sonora Margin	6/16/2010	1573	N 27 35.5827	W 111 28.9848	
12	1755-11	CT2	1–1.5	Cold seep, Vasconcelos active site	Sonora Margin	6/16/2010	1573	N 27 35.5820	W 111 28.9832	
13	1755-11	CT2	6–7							
14	1764-20	CT3	0–2	Hydrothermal vent, Mat Mound active site, orange mat	Southern Trough	6/27/2010	2005	N 27 00.3772	W 111 24.5641	Callac et al., under review
15	1766-22	CT2	0–5	Hydrothermal vent, MegaMat M27 active site, white mat	Southern Trough	6/29/2010	2003	N 27 00.4461	W 111 24.5243	
16	1766-22	CT2	5–10.5							
17	1753-09	CT4	8–11.5	Reference zone		6/14/2010	1850	N 27 25.4835	W 111 30.0779	Vigneron et al., 2013

### Chemical analyses

Pore water from the cores was extracted in one (<10 cm) or two centimeters (>10 cm) resolution using Rhizon moisture samplers with a pore size of 0.1 μm (Seeberg-Elverfeldt et al., 2005). Water samples were subsampled by adding either ZnCl_2_ (1:1 vol/vol) for H_2_S analysis or freezing at −20°C (ammonium) until further analysis. Ammonium was analysed by a manual flourimetric method (detection limit 1 μM, Holmes et al., 1999). This analysis is sensitive to the presence of hydrogen sulfide (H_2_S) therefore the measurements were corrected for the presence of H_2_S. Hydrogen sulfide concentrations were determined by colorimetry according to Fonselius et al. (2007).

### Molecular techniques

#### DNA isolation and polymerase chain reaction

Total genomic DNA was isolated from different sediments using the PowerSoil® DNA Isolation Kit (MO BIO Laboratories, Inc.) according to the manufacturer's protocol. To avoid contamination with our own anammox cultures, isolations were performed in a different department.

All PCR amplifications were performed in a total volume of 25 μl using 12.5 μl PerfeCTa® SYBR® Green FastMix (Quanta), 0.4 μM forward/reverse primer, 2 μl of template and 10.5 μl of DEPC-treated H_2_O. Hydrazine synthase amplification was initiated with a denaturation step at 94°C for 5 min and continued with a standard amplification program of 35 cycles (45 s 94°C; 1 min 56°C; 45 s 72°C). The final elongation step was done at 72°C for 7 min. Two different primer combinations, targeting the single copy *hzs*A gene, were used on the different samples (Harhangi et al., 2012): I (526F-TAYTTTGAAGGDGACTGG; 1857R-AAABGGYGAATCATARTGGC) and V (757F_Scal_-AGTTCNAAYTTTGACCC; 1857R-AAABGGYGAATCATARTGGC). The *aprA* fragments of 387 bp were amplified using primers aprA-1-FW-TGGCAGATCATGATYMAYGG and aprA-RV-GGGCCGTAACCGTCCTTGAA in the same assay as described for hydrazine synthase (Meyer and Kuever, 2007). The annealing temperature was lowered to 55°C.

#### Cloning and phylogenetic analysis

The *hzsA* and *aprA* amplicons were cloned in *Escherichia coli* using the pGEM T-easy vector system (Promega) according to the protocol. Clones were randomly selected from overnight-grown LB agar plates containing 100 μM of ampicillin, 200 μM IPTG and 200 μM X-gal. Plasmids were isolated with the GeneJet Plasmid Miniprep Kit (Thermo Fisher Scientific) according to the protocol using 4 ml of overnight bacterial culture. Colony PCR was performed to check cloned plasmids for an insert. Eighty *hzsA* clones and forty *aprA* clones were selected for Sanger sequencing at the DNA Diagnostics Department of Nijmegen University Medical Center, Nijmegen. Alignments and phylogenetic analysis were performed using the MEGA 5.0 software (Tamura et al., 2011). Related sequences were retrieved via BLAST searches in the Genbank databases. The sequences were submitted to Genbank under the accession numbers KF202916-KF202955 for *aprA* and KF202956-KF203035 for *hzsA* (see Supplementary Tables 1, 2).

#### Quantitative PCR

Primers targeting the *hzsA* gene (1600F_Scal_-GGKTATCARTATGTAGAAG; 1857R-AAABGGYGAATCATARTGGC) were used in a quantitative PCR assay to assess the absolute number of anammox bacteria in the deep sea samples. Amplifications were again performed in a total volume of 25 μl using iQ™ sybr® Green Supermix (Bio Rad) and 1 μl template. The amplicon of “*Ca*. Scalindua profunda” with a fragment amplified with the same primers (van de Vossenberg et al., 2013) was diluted in 10-fold steps and used as a standard in the analysis. Amplification was done on a iCycler iQ (Bio Rad) according to the following thermal protocol: The amplification program was started with 3 min at 96°C, followed by 40 cycles of 1 min at 95°C, 1 min 54°C, and 1 min at 72°C and a final elongation step of 5 min at 72°C. A melting curve analysis was performed at the end of the program ranging from 52–90°C in steps of 0.5°C to identify potentially unwanted amplicons. Ten products that were retrieved were cloned into *E. coli* as described above to verify amplification of the correct product. Retrieved plasmids were checked for an insert by colony PCR and sequenced as described before.

#### Lipid analysis

Ladderane fatty acids, including the newly identified short-chain C_14_ ladderane fatty acids, were analyzed according to previously described methods (Hopmans et al., 2006; Rush et al., 2012). Briefly, sediments were freeze-dried, homogenized, and extracted using a modified Bligh-Dyer method (Boumann et al., 2006). The extract was saponified by refluxing with aqueous 1 N KOH in 96% methanol for 1 h. The pH of the saponified extract was adjusted to 3 with 2 N HCl in methanol and the fatty acids were extracted with dichloromethane (DCM). The DCM fraction was dried using Na_2_SO_4_ and the fatty acids were converted into their corresponding fatty acid methyl esters (FAMEs) by methylation with diazomethane (CH_2_NH_2_). A FAME fraction was obtained by elution over activated aluminum oxide with DCM. Polyunsaturated fatty acids (PUFAs) were removed by elution over a small AgNO3 (5%)-impregnated silica column with DCM. The resulting fraction was dissolved in acetone (1 mg/ml) and filtered through a 0.45 μm, 4 mm diameter polytetrafluoroethylene (PTFE) filter and analyzed by high performance liquid chromatography coupled to positive-ion atmospheric pressure chemical ionization tandem mass spectrometry (HPLC/APCI-MS/MS) in selected reaction monitoring (SRM) mode as described by Rush et al. (2012). Ladderane FAMEs were quantified using external calibration curves of isolated methylated ladderane fatty acid standards.

## Results

### Biogeochemical analysis

**Figure 1:**
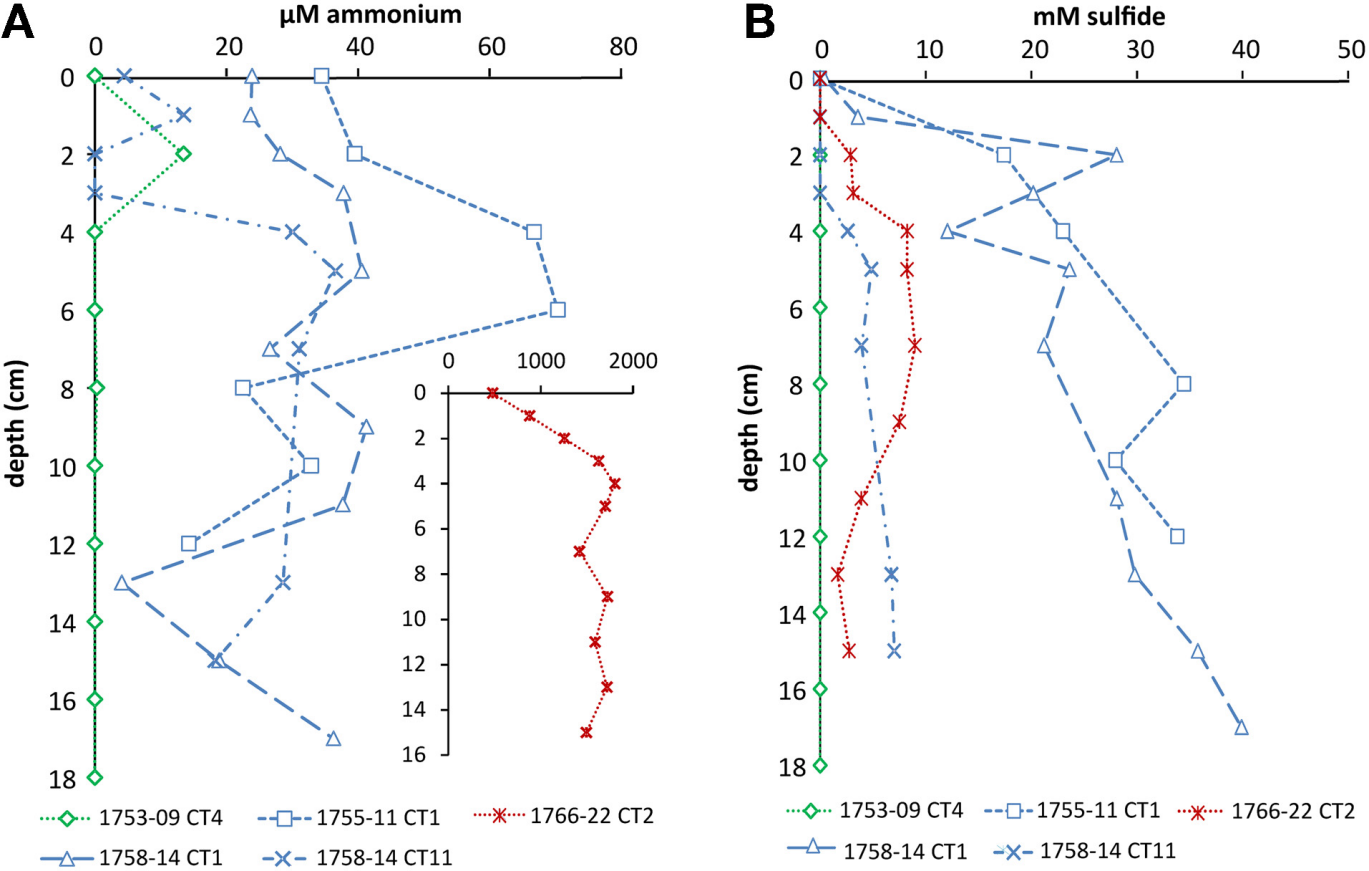
**Depth profiles of (A) ammonium and (B) sulfide in cold seep and hydrothermal sediments**. Sediment cores of the reference zone is depicted in green, cold hydrocarbon-rich seeps in blue and hydrothermal sites in red. Ammonium in hydrothermal sediments was higher and is therefore shown as an inset (the axes have the corresponding units).

Cold hydrocarbon-rich seep (CS) sediments were generally anoxic (Vigneron et al., 2013) and until a depth of 40 cm the temperature was constant at 3°C. As expected, ammonium was present throughout the sediment in concentrations ranging from 10–40 μM. In one core (1758-14 CT11) ammonium levels dropped below the detection limit (1 μM) at 2–3 cm, but increased again with increasing depth (Figure 1A). In sediments with hydrothermal activity ammonium concentrations were higher, increasing from 0.5 mM at the interface to 1.8 mM at 4 cm depth. In deeper layers, concentrations varied between 1.4 and 1.8 mM (Figure 1A). The temperature at the sediment-water interface was around 20°C and increased linearly to up to 100°C at 40 cm depth. Sulfide was not detected at the sediment interface in any of the samples, but increased rapidly below 1–4 cm sediment depth (Figure 1B). In the hydrothermal vent sediment and sediments at the edge of the white mat (1767 CT10 and 1758-14 CT11) concentrations stayed below 10 mM, whereas in sediments visibly covered by mats of sulfur oxidizers (1755-11 CT1 and 1758-14 CT1) sulfide concentrations were higher (Figure 1B). Within the reference zone (REF) core, which was collected outside of the active zone, there was a small peak of ammonium at 2 cm (13 μM). Sulfide was below detection limit throughout the core. Nitrate and nitrite profiles were not available for the sampling sites.

### Ladderane core lipid analysis

To confirm the presence and abundance of anammox bacteria in Guaymas Basin sediments we used ladderane fatty acids as an additional, specific biomarker. Selected samples were analyzed for original ladderane lipids (C_18_ and C_20_ ladderane fatty acids) as well as ladderane oxidation products (C_14_ ladderane fatty acids). The concentration of total ladderanes was highest in the cold hydrocarbon-rich seep sediments (up to 310 ng · g sediment^−1^) (Table 2). The relative contribution of short chain ladderane fatty acids to the total ladderane lipid pool in cold hydrocarbon-rich seep samples was 66 and 74% at the sediment-water interface (samples 1 and 12, respectively) and increased with sediment depth. The reference zone concentrations of ladderane fatty acids were 60 ng · g sediment^−1^ of which 80% were original ladderane lipids. In both hydrothermally active sediments (samples 14 and 15) no ladderane fatty acids could be detected.

**Table 2:** **Concentrations of total ladderane fatty acids in different sediment samples and the relative proportion of short chain ladderane fatty acids**.

**Sample number**	**1**	**3**	**6**	**9**	**11**	**12**	**13**	**14**	**15**	**17**
Ladderane fatty acids (ng · g sediment^−1^)	158	253	234	59	41	157	308	0	0	60
% short chain ladderane fatty acids	74	84	86	86	45	66	75	–	–	20

### Diversity of the *hzsA* gene in Guaymas Basin sediments

**Figure 2:**
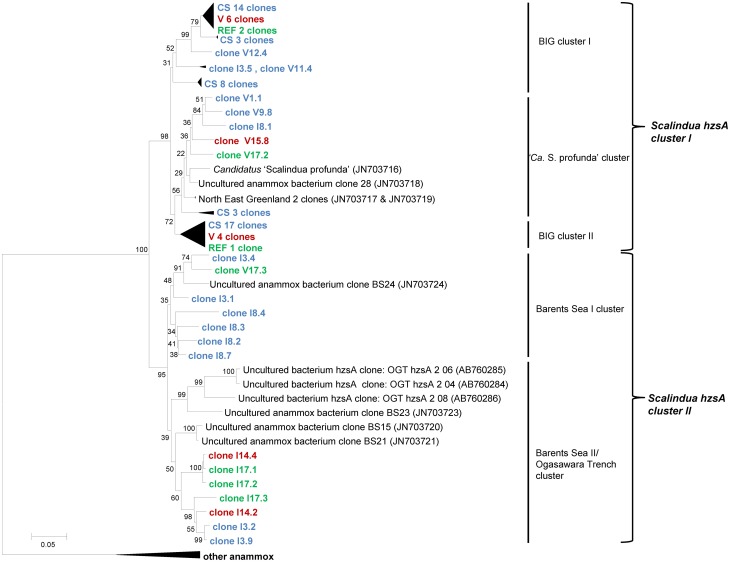
**Neighbor-joining tree of phylogeny estimated by ClustalW included in the MEGA 5.0 software package, showing >1000 bp fragments of *hzsA* nucleotide sequences retrieved from the Guaymas Basin sediments**. Letters (I or V) of the samples indicate the primer set used for amplification and the number refers to the sample. Samples are color-coded and at collapsed nodes abbreviated: reference zone in green (REF), cold hydrocarbon-rich seeps in blue (CS) and hydrothermal sites in red (V). Values at the internal nodes indicate bootstrap values based on 500 iterations. The bar indicates 5% sequence divergence. The outgroup with other anammox bacteria includes Genbank accession numbers JN703715, JN703714, JN703713, JN703712, AB365070 and CT573071. Accession numbers of individual clones are provided in Supplementary Table 1.

Amplifying the *hzsA* gene with two different primer sets (757F_Scal_/1857R and 526F/1857R) resulted in 80 clones from Guaymas Basin sediments, all of which were related to the “*Ca*. Scalindua” genus (Figure 2). Phylogenetic analysis revealed two distinct clusters of *hzsA* sequences (“*Ca*. Scalindua” *hzsA* cluster I and II). Sequences of cluster II were preferentially amplified using 526F/1857R as primers during amplification. The average similarity within these clusters was 91.6% for cluster I and 88.7% for cluster II. However, comparison of individual sequences revealed that between both clusters several sequences share a similarity as low as 76% at the nucleotide level. Within the “*Ca*. Scalindua” *hzsA* cluster I three subclusters could be identified: BIG I and BIG II consisted mostly of cold hydrocarbon-rich seep sequences. The third cluster consisted of sequences that were related to the enrichment culture species *S. profunda* (van de Vossenberg et al., 2008) as well as three clones from Northeast Greenland marine sediment (Harhangi et al., 2012). The “*Ca*. Scalindua” hzsA cluster II was generally more diverse and composed of two subclusters. The first subcluster containing one sequence from Barents Sea sediment and mostly sequences associated with cold hydrocarbon-rich seeps. The second cluster contained several clones retrieved from the Barents Sea (Harhangi et al., 2012), clones from the Ogasawara Trench in the West Pacific (Nunoura et al., 2013) and clones from cold hydrocarbon-rich seeps, vents and reference zone samples. Generally there seems to be no great correlation between the sample locations and the diversity of anammox bacteria as clones retrieved from cold hydrocarbon-rich seep, reference zone and hydrothermal sediments were found in all clusters.

### Quantification of anammox bacteria by functional gene amplification

**Figure 3:**
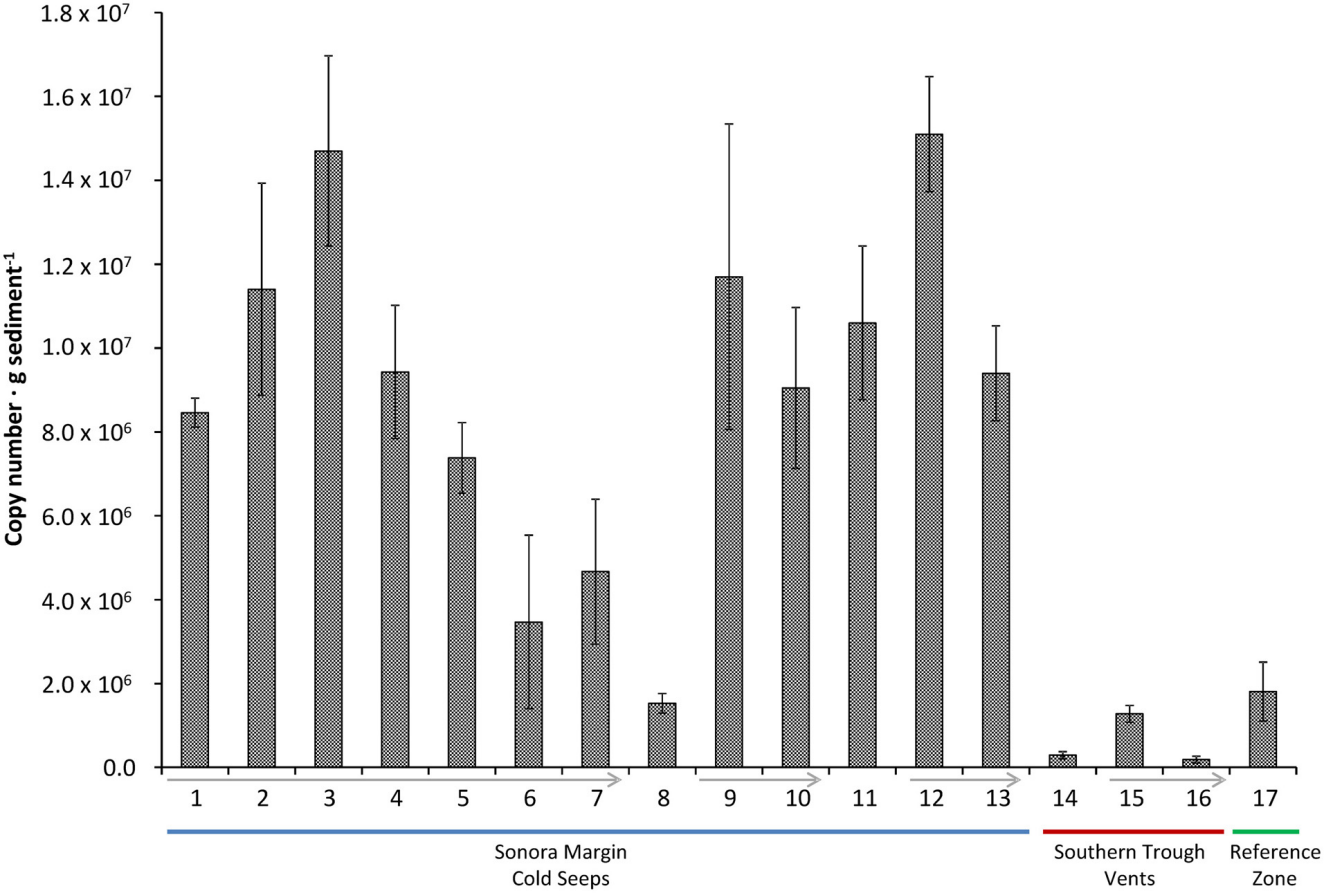
***hzsA* copy number in different sediment samples (±*SD* of technical replicates)**. Sediment cores of the reference zone is depicted in green, cold hydrocarbon-rich seeps in blue and hydrothermal sites in red. Arrows indicate decreasing sediment depth within a single core.

Based on the *hzs*A biodiversity study (see above) which showed only representatives of the “*Ca*. Scalindua” genus, we determined absolute numbers of anammox bacteria in the sediment samples by amplification of a “*Ca*. Scalindua”-specific 257 bp fragment of the *hszA* gene in a qPCR assay. The results indicated that numbers of anammox bacteria were generally higher in cold hydrocarbon-rich seep environments compared to the vents (Figure 3).

Gene copy numbers of anammox *hzsA* in cold hydrocarbon-rich seep sediments varied between 1.5 × 10^6^ to 1.5 × 10^7^ copies per gram of sediment. The total number of *hzsA* gene copies was usually highest at the sediment interface and decreased with increasing depth (Figure 3, 9/10, 12/13, 15/16). The exception was a whole sediment core (Figure 3, 1–7) (0–21 cm), which was analyzed in increments of 3 cm. Here, *hzsA* gene copies increased linearly from the interface peaking at 6–9 cm sediment depth (1.47 × 10^7^ copies · g sediment^−1^). In proximity of the hydrothermal vents total numbers of the *hzsA* gene in the sediments were much lower (1.9 × 10^5^ to 1.3 × 10^6^ copies · g sediment^−1^).

### Diversity of the *aprA* gene

**Figure 4:**
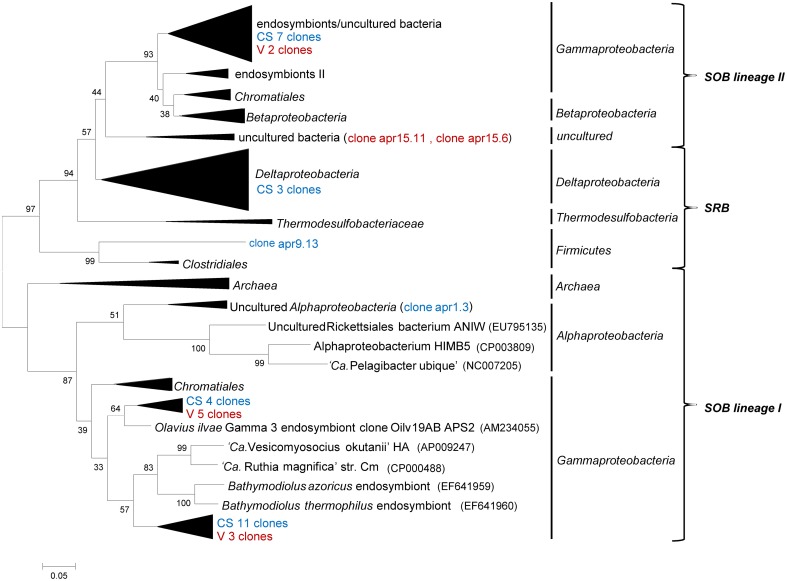
**Neighbor-joining tree of phylogeny estimated by ClustalW included in the MEGA 5.0 software package, showing ~400 bp fragments of *aprA* nucleotide sequences retrieved from the Guaymas Basin sediments**. The number refers to the sample and collapsed nodes are abbreviated: reference zone in green (REF), cold hydrocarbon-rich seeps in blue (CS) and hydrothermal sites in red (V). Values at the internal nodes indicate bootstrap values based on 500 iterations. The bar indicates 5% sequence divergence. Accession numbers of individual clones are provided in Supplementary Table 2.

An *aprA* library comprising 39 sequences was generated from selected cold hydrocarbon-rich seep sediments (samples 1, 3, and 9) and the sediment interface of the vents MegaMat M27 (sample 15). The majority of all sequences were affiliated with the *Gammaproteobacteria* (35 sequences), clustering in both *apr* lineages of known sulfur oxidizing bacteria (SOB) (Meyer and Kuever, 2007). Sequences from lineage I were divided into 2 clusters (Figure 4). The first cluster consisting of 4 cold hydrocarbon-rich seep clones and 5 from hydrothermal vent sediments were most closely related to gut microflora clones of *Asterechinus elegans* (93%) and endosymbionts of *Olavius ilvae* (86–89%) (Ruehland et al., 2008; Becker et al., 2009). The second cluster was comprised of 11 cold hydrocarbon-rich seep clones and 3 vent clones showing the highest similarity to clones retrieved from hydrothermal vents of the Logatchev field (87–89%) and low temperature hydrothermal oxides at South West Indian ridge (90%) (Hügler et al., 2010). One clone was retrieved within lineage I being most closely related with the uncultured alphaproteobacterial *aprA* genes. The closest hit was a clone retrieved from carbonate sediments at the South West Indian Ridge (88%). Within SOB *apr* lineage II 9 sequences (7 CS and 2 V) grouped within a cluster of endosymbionts and uncultured bacteria of deep-sea environments. Closest hits were obtained with marine sediment clones of the Cascadia margin (94%), gut microflora clones of *Asterechinus elegans* (92%) and endosymbionts of *Riftia pachyptila* (86%) (Meyer and Kuever, 2007; Becker et al., 2009; Blazejak and Schippers, 2011; Brissac et al., 2011; Lenk et al., 2011). Two clones from vent sediments formed a separate cluster in *apr* lineage I, showing a rather low similarity to previously described clones (gut microflora clones of *Asterechinus elegans* 81%). Three of the *aprA* sequences clustered with sulfate-reducing *Deltaproteobacteria* of the *Desulfovibrio* and *Desulfobulbus* genus and a single sequence was most closely related with the firmicutes (*Desulfotomaculum sp*. 78%) (Friedrich, 2002; Hügler et al., 2010).

## Discussion

**Figure 5:**
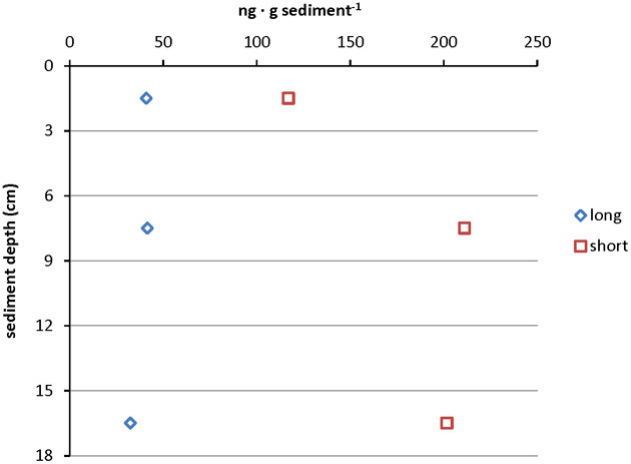
**Depth profile of absolute ladderane lipid concentrations in core 1758-14 CT2 including original ladderanes and short chain ladderane fatty acids**. Data points represent measurements on the sediment sample pooled from 0 to 3 cm, 6 to 9 cm, and 15 to 18 cm sediment depth.

Almost all samples from the cold hydrocarbon-rich seep sediment core that were investigated contained a high relative abundance of short chain ladderane fatty acids (>65%). This high percentage may be explained by degradation of original ladderanes during diagenesis (Rush et al., 2012). Little is known about the biological degradation of ladderanes yet, but it is assumed that it proceeds via the β-oxidation pathway (Beam and Perry, 1974; Dutta and Harayama, 2001). Although the sediments were virtually anoxic, the degradation of original ladderane fatty acids could be the result of periodic exposure to low amounts of oxygen in such a dynamic system. When the concentrations of short and original ladderane fatty acids were compared, a slight increase of original ladderanes and an increase in short chain ladderanes with depth was observed, which corresponded to the acquired qPCR data of core 1758-14 CT2 (Figure 5). This would suggest that on the one hand there was ongoing conversion from long chain fatty acids to the short chain ladderanes in the absence of oxygen, but also that the overall concentration of original ladderanes was higher deeper in the sediment at this specific location. This could be the result of seasonal high burial of anammox biomass and a high ladderane turnover or the *in situ* production of ladderanes in deeper sediment layers. In hydrothermally active sediments no ladderanes could be detected. This could be because ladderanes were unstable at higher temperatures due to their peculiar chemical structure (Jaeschke et al., 2008).

The use of two different primer sets to amplify *hzsA* gene fragments resulted in the retrieval of marine *hzsA* sequences showing a diversity comparable to that reported for 16S rRNA genes of anammox bacteria (Woebken et al., 2008). The functional gene analysis hinted at the high diversity within the “*Ca*. Scalindua” genus, considering that some genera of fresh water anammox shared a higher similarity (*Ca*. Jettenia asiatica and *Ca*. Brocadia anammoxidans 79.4%). This was a first indication using a functional gene that the marine group might consist of more than one genus or at least showed a very high interspecies diversity within a single genus.

The finding of anammox specific phylomarkers within these sediments was surprising, as the deep sea sediments of the Guaymas Basin were not only rich in organic matter, ammonium and methane, but also contained mM concentrations of sulfide. Sulfide has been reported to inhibit the anammox process in granular sludge of wastewater treatment plants already at low concentrations (Carvajal-Arroyo et al., 2013; Jin et al., 2013). Although no conclusion can be made with regard to anammox activity in the Guaymas Basin sediments, the results point to the same direction as the highest number of *hzsA* copy numbers usually coincided with the absence of sulfide at the sediment interface and decreased rapidly with depth.

Aerobic ammonium oxidizing Thaumarchaeota were shown to play a role in supplying nitrite for anammox in oxygen minimum zones (OMZ) (Francis et al., 2005; Lam et al., 2009), but in these systems also a cryptic sulfur cycle was reported (Canfield et al., 2010). Since for continental shelf sediments and the Benguela OMZ denitrification was suggested to play a role in nitrite supply (Thamdrup and Dalsgaard, 2002; Kuypers et al., 2005) and the sites of our study are all sulfidic in nature we focused on the possibility of sulfide-driven partial denitrification. Reduced sulfur compounds, such as sulfide, fuel primary production in cold hydrocarbon-rich seeps and are often linked to oxygen and nitrate respiration (Jannasch and Wirsen, 1979; Karl et al., 1980; Lichtschlag et al., 2010). The amplification of a key gene in sulfur oxidation revealed a high number of gammaproteobacterial *aprA* genes covering the two SOB *aprA* lineages as well as sulfate-reducers. Previously also *Epsilonproteobacteria* were shown to be abundant in Guaymas Basin sediments likely also gaining energy from growth on sulfide as an electron donor to reduce nitrate (Teske et al., 2002; Bowles et al., 2012). This suggested that reduced sulfur compounds could serve as a link between sulfur and nitrogen cycling in such ecosystems. Although there was no direct evidence for the significance of *Gammaproteobacteria* in sulfur oxidation in the Guaymas Basin sediments the retrieval of such an *aprA* diversity confirmed findings of *Gammaproteobacteria* playing a role in linking the sulfur and nitrogen cycles in marine sediments by coupling sulfide oxidation to nitrate reduction (Mills et al., 2004; Hügler et al., 2010; Lenk et al., 2011). This could have very interesting implications with respect to the formation of complex interactions that would be driven by the exchange of intermediates (i.e., nitrite). For example, partial denitrification (Błaszczyk, 1992, unpublished data) coupled to sulfide oxidation could supply anammox bacteria with nitrite. Additionally, the oxidation of sulfide might create pockets in which the concentration of free sulfide is low enough so that anammox bacteria remain active. The existence of such an interaction was recently reported (Wenk et al., 2013), but whether this could occur in the sediments or the water column of the Guaymas Basin remains to be determined.

## Conclusion

This study shows that anammox bacteria were detected in complex and exotic environments by amplification of the unique functional marker gene *hzsA*, allowing much more specificity than 16S rRNA gene based analysis. The high diversity observed in *hzsA* phylogeny suggested a high interspecies variety within the marine anammox cluster in an essential and highly-conserved gene. Although evidence so far did not favor anammox bacteria in sulfidic sediments (Burgin and Hamilton, 2007) we detected relatively high numbers of anammox gene copies in cold hydrocarbon-rich seep sediments of the Guaymas Basin.

### Conflict of interest statement

The authors declare that the research was conducted in the absence of any commercial or financial relationships that could be construed as a potential conflict of interest.
