# Supplementary material for: Presence and diversity of anammox bacteria in cold hydrocarbon-rich seeps and hydrothermal vent sediments of the Guaymas Basin
Source: Front Microbiol. 2013 Aug 2;4:219. doi: 10.3389/fmicb.2013.00219 (PMC3731535; doi:10.3389/fmicb.2013.00219)
Supplement: Supplementary file 1 [file 52755_Op_Den_Camp_DataSheet1.PDF]

Cluster	Position in tree	No. used in the article	No. in Genbank	Accession no.
BIG cluster I	CS 14 clones	V8.1	V12.1	KF203008
		V9.4	V28.4	KF203030
		V1.2	V1.2	KF202988
		V9.6	V28.6	KF203032
		V8.2	V12.2	KF203009
		V9.1	V28.1	KF203027
		V12.1	V8.1	KF202991
		V9.3	V28.3	KF203029
		V1.4	V1.4	KF202990
		V12.2	V8.2	KF202992
		V8.5	V12.5	KF203012
		V9.2	V28.2	KF203028
		I3.3	I3.3	KF202961
		V12.7	V8.7	KF202997
	V 6 clones	V15.5	V24.5	KF203022
		I14.3	I22.3	KF202982
		V14.3	V24.3	KF203020
		I14.1	I22.1	KF202980
		V15.7	V24.7	KF203024
		V15.9	V24.9	KF203026
	REF 2 clones	V17.8	V23.8	KF203017
		V17.1	V23.1	KF203013
		V12.4	V8.4	KF202994
	CS 3 clones	V9.5	V28.5	KF203031
		I1.1	I1.1	KF202956
		I3.11	I3.11	KF202968
		V8.4	V12.4	KF203011
		I3.5	I3.5	KF202963
		V11.4	V11.4	KF203001
	CS 8 clones	V11.8	V11.8	KF203005
		V9.7	V28.7	KF203033
		V11.10	V11.10	KF203007
		I11.1	I11.1	KF202971
		I11.2	I11.2	KF202972
		V12.5	V8.5	KF202995
		V11.2	V11.2	KF202999
		V11.3	V11.3	KF203000
	CS 17 clones	V12.6	V8.6	KF202996
		V11.1	V11.1	KF202998
		I1.2	I1.2	KF202957
		V11.7	V11.7	KF203004
		I3.7	I3.7	KF202965
		V8.3	V12.3	KF203010
		I3.1	I3.1	KF202959

BIG cluster II		V11.6	V11.6	KF203003
		I12.1	I8.1	KF202969
		V1.3	V1.3	KF202989
		I1.3	I1.3	KF202958
		I12.2	I8.2	KF202970
		I3.6	I3.6	KF202964
		V12.3	V8.3	KF202993
		I8.5	I12.5	KF202977
		V12.4	V8.4	KF202994
		V9.9	V28.9	KF203035
	V 4 clones	V15.4	V24.4	KF203021
		V15.6	V24.6	KF203023
		V15.1	V24.1	KF203018
		V15.2	V24.2	KF203019
	REF 1 clone	V17.6	V23.6	KF203016
Barents Sea I		I3.4	I3.4	KF202962
		V17.3	V23.3	KF203015
		I3.1	I3.1	KF202959
		I8.4	I12.4	KF202976
		I8.3	I12.3	KF202975
		I8.2	I12.2	KF202974
		I8.7	I12.7	KF202978
Barents Sea II		I14.4	I22.4	KF202983
		I17.1	I23.1	KF202984
		I17.2	I23.2	KF202985
		I17.3	I23.3	KF202986
		I14.2	I22.2	KF202981
		I3.2	I3.2	KF202960
		I3.9	I3.9	KF202967
'*Ca*. S. profunda' cluster		V1.1	V1.1	KF202987
		V15.8	V24.8	KF203025
		I8.1	I12.1	KF202973
		V9.8	V28.8	KF203034
		V17.2	V23.2	KF203014
	CS 3 clones	I3.8	I3.8	KF202966
		V11.5	V11.5	KF203002
		V11.9	V11.9	KF203006

Supplementary Table 1: Description of the *hzsA* clones used to make the phylogenetic tree, including the numbering used in this article and the numbering used in Genbank as well as the accession number for each clone.
