# Supplementary material for: Presence and diversity of anammox bacteria in cold hydrocarbon-rich seeps and hydrothermal vent sediments of the Guaymas Basin
Source: Front Microbiol. 2013 Aug 2;4:219. doi: 10.3389/fmicb.2013.00219 (PMC3731535; doi:10.3389/fmicb.2013.00219)
Supplement: Supplementary file 2 [file 52755_Op_Den_Camp_DataSheet2.PDF]

Cluster	Order	Position in tree	No. used in the article	No. in Genbank	Accession no.
SOB lineage II	*Gammaproteobacteria*	CS 7 clones	apr3.1	apr3.1	KF202925
			apr3.4	apr3.4	KF202923
			apr1.10	apr1.10	KF202952
			apr9.2	apr28.2	KF202937
			apr1.9	apr1.9	KF202953
			apr1.4	apr1.4	KF202918
			apr9.7	apr28.7	KF202932
	uncultured	V 2 clones	apr15.11	apr24.11	KF202940
			apr15.6	apr24.6	KF202945
SRB	*Deltaproteobacteria*	CS 3 clones	apr9.11	apr28.11	KF202928
			apr1.8	apr1.8	KF202954
			apr9.3	apr28.3	KF202936
	Firmicutes		apr9.13	apr28.13	KF202926
SOB lineage I	*Alphaproteobacteria*		apr1.3	apr1.3	KF202919
	Gammaproteobacteria	CS 4 clones	apr9.8	apr28.8	KF202931
			apr9.5	apr28.5	KF202934
			apr1.2	apr1.2	KF202920
			apr9.6	apr28.6	KF202933
		V 5 clones	apr15.2	apr24.2	KF202949
			apr15.9	apr24.9	KF202942
			apr15.7	apr24.7	KF202944
			apr15.12	apr24.12	KF202939
			apr15.10	apr24.10	KF202941
		CS 11 clones	apr9.3	apr28.3	KF202936
			apr9.12	apr28.12	KF202927
			apr1.1	apr1.1	KF202921
			apr3.5	apr3.5	KF202922
			apr1.5	apr1.5	KF202917
			apr1.7	apr1.7	KF202955
			apr9.4	apr28.4	KF202935
			apr9.9	apr28.9	KF202930
			apr3.3	apr3.3	KF202924
			apr1.6	apr1.6	KF202916
			apr1.11	apr1.11	KF202951
			apr9.10	apr28.10	KF202929
		V 3 clones	apr15.3	apr24.3	KF202948
			apr15.1	apr24.1	KF202950
			apr15.8	apr24.8	KF202943

Supplementary Table 2: Description of the *aprA* clones used to make the phylogenetic tree, including the numbering used in this article and the numbering used in Genbank as well as the accession number for each clone. The order of clones corresponds to the organization of the phylogenetic tree.
